# Discovery of the allosteric inhibitor from actinomyces metabolites to target EGFR^CSTMLR^ mutant protein: molecular modeling and free energy approach

**DOI:** 10.1038/s41598-023-33065-7

**Published:** 2023-06-01

**Authors:** Ravi Saini, Sonali Kumari, Aditi Bhatnagar, Amit Singh, Abha Mishra

**Affiliations:** 1 School of Biochemical Engineering, Indian Institute of Technology (BHU), Varanasi, 221005 India; 2 Department of Pharmacology, Institute of Medical Sciences, Banaras Hindu University, Varanasi, 221005 India

**Keywords:** Cancer, Computational biology and bioinformatics, Drug discovery

## Abstract

EGFR (epidermal growth factor receptor), a surface protein on the cell, belongs to the tyrosine kinase family, responsible for cell growth and proliferation. Overexpression or mutation in the EGFR gene leads to various types of cancer, i.e., non-small cell lung cancer, breast, and pancreatic cancer. Bioactive molecules identified in this genre were also an essential source of encouragement for researchers who accomplished the design and synthesis of novel compounds with anticancer properties. World Health Organization (WHO) report states that antibiotic resistance is one of the most severe risks to global well-being, food safety, and development. The world needs to take steps to lessen this danger, such as developing new antibiotics and regulating their use. In this study, 6524 compounds derived from *Streptomyces* sp. were subjected to drug-likeness filters, molecular docking, and molecular dynamic simulation for 1000 ns to find new triple mutant EGFR^CSTMLR^ (EGFR-L858R/T790M/C797S) inhibitors. Docking outcomes revealed that five compounds showed better binding affinity (− 9.074 to − 9.3 kcal/mol) than both reference drug CH7233163 (− 6.11 kcal/mol) and co-crystallized ligand Osimertinib (− 8.07 kcal/mol). Further, molecular dynamic simulation confirmed that ligand C_42 exhibited the best interaction at the active site of EGFR protein and comprised a better average radius of gyration (3.87 Å) and average SASA (Solvent Accessible Surface Area) (82.91 Å2) value than co-crystallized ligand (4.49 Å, 222.38 Å2). Additionally, its average RMSD (Root Mean Square Deviation) (3.25 Å) and RMSF (Root Mean Square Fluctuation) (1.54 Å) values were highly similar to co-crystallized ligand (3.07 Å, 1.54 Å). Compared to the reference ligand, it also demonstrated conserved H-bond interactions with the residues MET_793 and GLN_791 with strong interaction probability. In conclusion, we have found a potential drug with no violation of the rule of three, Lipinski's rule of five, and 26 other vital parameters having great potential in medicinal and pharmaceutical industries applications and can overcome synthetic drug issues.

## Introduction

Cancer is one of the primary reasons for mortality worldwide, and its prevalence continuously increases. According to GLOBOCAN 2020, about 10 million people died from cancer in 2020, and 19.3 million new cases appeared worldwide^1^. Cancer in the lungs, prostate, colon, and urinary bladder are most common in men, while breast, cervical, and uterine cancer frequently occur in women^2^.

EGFR (epidermal growth factor receptor) is a tyrosine kinase family's 170 kDa transmembrane glycoprotein. It is mainly a cell surface receptor containing three parts: intracellular tyrosine kinase, an extracellular ligand-binding domain, and a transmembrane anchoring region^3,4^. It binds to epidermal growth factor, which leads to its dimerization and autophosphorylation of tyrosine residues, which activate a cascade of intracellular signal transduction events and promote cell proliferation, angiogenesis, apoptosis resistance, cellular invasion, and metastasis^5^.

Mutation or overexpression of the EGFR gene causes different cancers type, including breast cancer, prostate cancer, ovarian cancer, and NSCLC (non-small-cell lung cancer)^6^. Five amino acids (codons 746 to codon 750) are responsible for point mutation of EGFR at the L858 position (catalytic domain of EGFR) and cause autophosphorylation and unregulated cell growth. Gefitinib and erlotinib (first-generation EGFR tyrosine kinase inhibitors) are reversible inhibitors that compete with ATP (Adenosine triphosphate) to bind at the catalytic site of EGFR and cause hindrance of autophosphorylation and cell proliferation^7^. The need for inhibitors keeps increasing; consequently, the effectiveness of first-generation inhibitors was hindered due to resistance raised for these inhibitors, which diminished the clinical response over a year. Substitution of threonine 790 with methionine, EGFR^T790M^ (EGFR-L858R/T790M) is one of the most frequent resistance issues against first-generation tyrosine kinase inhibitors and causes a reduction in its inhibitor's activity and prevents their interaction at the catalytic domain^8^. Approximately 50% of NSCLC-affected patients with mutated EGFR^T790M^ (EGFR-T790M) were resistant to first-generation tyrosine kinase inhibitors. Thus, the alteration in EGFR at T790M established the development of resistance against first and second-generation tyrosine kinase inhibitors and provoked the finding of inhibitors of the third-generation kinase. Neratinib, Dacomitinib, and Afatinib are second-generation kinase inhibitors that interact covalently with Cys_797 residue at the binding pocket of ATP, and their clinical application is restricted because of side effects such as gastrointestinal problems and skin diseases^9,10^.

Third-generation kinase inhibitors showed significant efficiency in NSCLC-affected patients possessing resistance to first/second-generation inhibitors. Osimertinib is a Food and Drug Administration (FDA)-approved third-generation inhibitor, expressing effective results in NSCLC patients^11^. Further, the development of fourth-generation kinase inhibitors comes into the limelight with the emergence of resistance to third-generation kinase inhibitors. The third-generation EGFR tyrosine kinase inhibitors, i.e., EGFR^CSTMLR^ (EGFR-L858R/T790M/C797S) resistance developed by converting cysteine to serine at 797, which is less reactive and inhibits the interaction between inhibitors and cysteine amino acid^12^. Numerous side effects are also associated with third-generation inhibitors, so an alternative drug based on natural sources, having fewer side effects and low cost, needs to be discovered^13^.

In this regard, *Streptomyces* can be a perfect alternative for discovering EGFR tyrosine kinase inhibitors due to their greater bioavailability, bountiful sources of secondary metabolites, biotic friendliness, lesser side effects, non-toxic and highly effectiveness^14^. Genus *Streptomyces* belongs to the phylum actinobacteria and is primarily ubiquitous, aerobic, Gram-positive, and filamentous soil bacteria^15^. They can produce many secondary metabolites due to the survival of their spores in adverse environmental conditions. Today, the genus Streptomyces is responsible for developing 80% of antibiotics, making it the most important genus in medication discovery against various diseases^16^. They also create complex secondary metabolites such as enzymes, protein kinase inhibitors, apoptosis inducers, and caspase-3 activators, which are helpful in cancer treatment. For example, Streptomyces verticillus synthesized the anticancer drug bleomycin^17^, which was FDA-approved in 1973, and has therapeutic potential in cervical carcinoma, lymphoma, head and neck cancer, and testicular cancer^18^.

In order to find inhibitors with a strong binding affinity and ideal ADMET (Absorption, Distribution, Metabolism, Excretion, and Toxicity) profile, molecular docking and dynamics simulation studies have recently been applied to develop new drugs^19–23^. Finding new potential EGFR^CSTMLR^ tyrosine kinase inhibitors from the StreptomeDB database is the goal of the current investigation.

## Methodology

### Retrieval and processing of ligand database

StreptomeDB is a curated database of Isolated or mutasynthesized Natural Products (NPs) from Streptomyces^24^. The StreptomeDB 3.0 is an updated database with 6524 NPs from 3302 actinomyces species^25^. The three-dimensional structures of these 6524 NPs were downloaded in SDF file format from http://www.pharmbioinf.uni-freiburg.de/streptomedb. All the NPs and reference molecules were converted into mae file format for easy processing by utilizing the LigPrep module of Schrödinger (Schrödinger, LLC, New York, USA, 2022). LigPrep module windows were set to OPLSe (Optimized Kanhesia for Liquid Simulations) forcefield, ion neutralization, and single isomers generation per ligand^26^. Reference structure CH7 (CH7233163) and co-crystal ligand Osimertinib of the target were used as the standard reference for the study^27,28^.

### Retrieval and processing of target

A 3-D crystal structure of EGFR^CSTMLR^ was downloaded from the RCSB-PDB (RCSB Protein Data Bank) with PDB id: 6LUD^27^. The receptor was already co-crystallized with a well-known anti-Tyrosine Kinase inhibitor: Osimertinib. The structure was then imported into the Protein preparation wizard of Schrödinger to rectify the problems in protein structure. The protein was further refined by optimizing hydrogen bonds and minimizing its structural energy with the OPLSe force field^26,29^. The receptor grid generation module in Glide-Schrödinger creates a grid box over the ligand with a Van-der-Waal scaling factor set to 1.0, and a partial charge cutoff is set to 0.25.

### ADME analysis of ligand library

ADME is a test that establishes the absorption, distribution, metabolism, and excretion characteristics of a potential principal compound and helps study its disposition within an organism^30^. ADME was performed by incorporating the QikProp program of Schrödinger running in standard default settings (Schrödinger, LLC, New York, USA, 2022). This program helps generate the relevant descriptor (set of 46 molecular descriptors) and uses selected descriptors to carry out ADMET predictions. Drug-likeness of a lead depends on various parameters, and they were used to estimate the pharmacokinetic analysis of the StreptomeDB ligand library. Selected 26 descriptors that play a significant role in most drug-likeliness criteria were chosen on the ligand library, making them match 95% of known drug ranges.

### Molecular docking simulations

Molecular docking was performed using Glide software from the Schrödinger suite with Standard-Precision protocol (SP) with ten poses included in post-docking minimization keeping all other parameters default^31^. Re-docking, the co-crystallized ligand with a suitable RMSD (Root Mean Square Deviation) value (less than 2 Å), enabled to verify the docking procedure. The re-docked poses gave away similar interactions compared to the active site with the native pose. The above experiment leads to the inference that the docking protocol is reliable and sustainable for predicting plausible inhibitors in the used StreptomeDB database^32^. For further evaluation, the compounds were selected based on the obtained glide score, binding energy, and analysis of the binding pattern of the ligand–protein complex. The PAINS (pan assay interference compounds) was also applied to the selected compound using the Knime Analytics Platform^33^ with PAINS, PAINS-A, PAINS-B, and PAINS-C filter criteria of RDKit Molecule catalog filter node.

### Molecular dynamics simulations

The enzyme–inhibitor complexes that had been docked were considered as the initial framework for future simulation runs. The Schrödinger Suite's Desmond simulation tool was used to run the MD (Molecular Dynamics) simulations (Schrödinger, LLC, New York, USA, 2022). The orthorhombic box was solvated using the TIP3P (transferable intermolecular potential with 3 points) solvation/aqueous system, and the solvent buffer was expanded around the protein by 10 Å using the OPLSe force field^34^. The system was then neutralized and maintained at 0.15 M salt concentration by placing the required NaCl counter ions. Before employing the simulation study, Desmond's pre-defined system relaxation algorithm was used to equilibrate the system, consisting of six phases in the protocol. The first two were set for the NVT simulation at 10 K temperature, where the first phase was carried out for 100 ps (picosecond) using the Brownian dynamics method to restrict the motion of heavy atoms present in the protein molecules, while in the second stage, the restriction was applied on the large atom of solute for next 12 ps. After that, the NPT condition was applied in the third step by restricting the atom of solute again at 10 K for 12 ps, and the constant pressure was almost one bar. The temperature was likely to be enhanced up to 310 K in the fourth stage with 10 ps time at NPT condition for relaxing the protein molecules and possessing no restriction on the system. The final simulation time was 1000 ns (nanosecond), with a 1 ns recording interval. All simulations used the OPLS4 force field parameters. To calculate the MM/GBSA (Molecular Mechanics and Generalized Bonn-Surface Area) representing the following equation, the prime module of Schrödinger was used at the last 100 ns of the MD trajectories:

$$\Delta {G}_{bind}={\Delta G}_{comp} - [{\Delta G}_{protein}+\Delta {G}_{lig}]$$

where ΔG_bind_ represents the free binding energy, ΔG_protein_ is the binding energy of the target protein, and ΔG_lig_ represents the binding energy of the ligand in the protein–ligand complex^35^.

## Results and discussion

### ADMET properties predictions

It has been suggested that the compounds used as human therapeutic agents should have drug-likeliness properties or exhibit good ADMET profiles^36^. The ADME properties of 6524 compounds were ascertained using the QikProp web service against selected 25 molecular descriptors criteria. QikProp program predicted that the potential to penetrate the blood/brain barrier and bind with human serum albumin was portrayed by 71.8% and 85% virtual hits. Ro5 (Lipinski's rule of five) for screening drug-like compounds showed that 76% of molecules retained physicochemical properties and followed Lipinski's parameters. In addition, the Ro3 (Ghose rule of three) was applied to enhance the ADME prediction and observed that 67% of molecules were passed through it.

**Figure 1:**
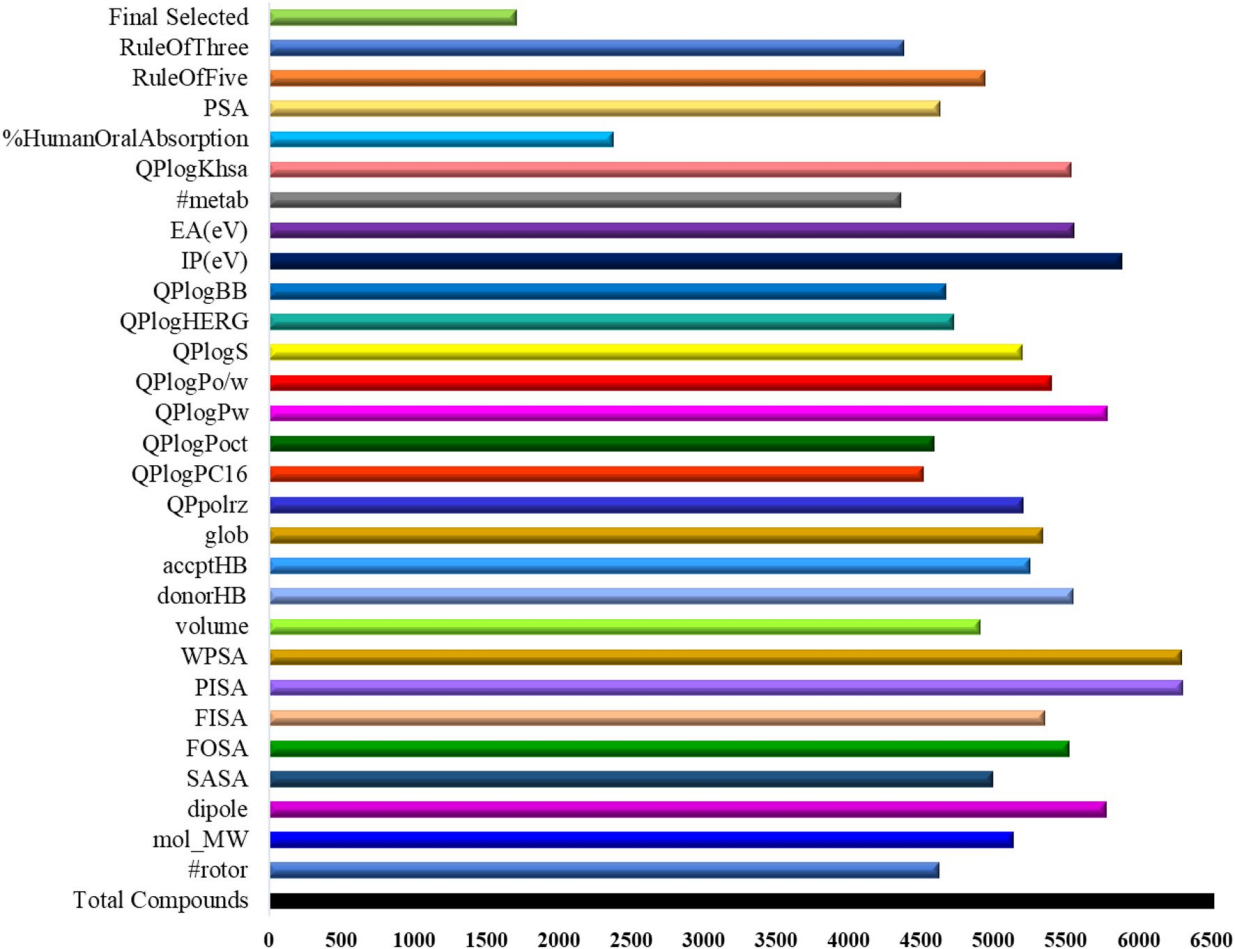
ADMET profile of 6524 compounds from StreptomeDB 3.0 database.

Further, the potassium ion (K^+^) channel linked to the Human ether-a-go-go-related gene (HERG) is responsible for long QT syndrome^37^. This channel plays a vital role in regulating heartbeats, making it a vulnerable target to the potential therapeutic drug for cardiac toxicity^38^. So, the value of predicted IC_50_ was calculated for the inhibitory action on HERG K^+^ channel by drugs to model drug toxicity effect, and it was found that both the standard drug lies far outside the safe threshold. 1708 (26.2%) compounds depicted a remarkable result by effectively passing through all the ADMET parameters and were selected for anticancer activity analysis by being subjected to the molecular docking study with the target protein EGFR-tyrosine kinase. The ADME properties of all 6524 compounds are presented in Fig. 1. All the compounds passed through the above criteria lie in the zero-violation category of Ro3 and Ro5 (except C_5024 with only one violation in Ro3), whereas both Osimertinib and CH7 have one violation in Ro3, 1 and 3 violations in Ro5, respectively.

### Molecular docking study of triple mutant EGFR^CSTMLR^

Molecular docking of 2124 compounds at the active site of mutant EGFR^CSTMLR^ leads to the identification of a novel potential drug for anticancer. Table 1 shows the hit compound's binding score, hydrogen, and hydrophobic bond-forming amino-acid residues. Out of 2124 compounds, five were screened out, which are C_42: Steptocarbazole A, C_4299: Limazepine H, C_4300: 3'N-Formylholyrine A, C_5024: CHEMBL1159781, and C_5702: 3′-*N*-acetylholyrine A, revealing a better binding score than reference inhibitor CH7: CH7233163 and co-crystallized ligand Osi: Osimertinib (well-known Tyrosine inhibitor). Duration of the interaction^39^. It was found that these selected molecules have also passed all criteria of the PAINS filter as well.

**Table 1:** Binding energy and molecular interaction analysis (Hydrogen and Hydrophobic interaction) ligands with triple mutant EGFR^L858R/T790M/C797S^ and their production source.

Ligands	Docking Score (Kcal/mol)	Hydrogen bond interaction	Hydrophobic interaction	Microbial source of production^†^	Compound ID
C_42	− 9.3	GLN_791, **MET_793**, ASP_800	LEU_718, PHE_723, **VAL_726**, **ALA_743**, **MET_790**, **LEU792, PRO_794, LEU_844**	*Streptomyces sanyensis* FMA	PubChem 72501071
C_4299	− 9.164	LYS_745, GLU762, **MET_793**	LEU_718, **VAL_726, ALA_743**, MET766, **MET_790**, **LEU_792, PRO_794, LEU_844,** PHE856	*Streptomyces seoulensis* IFB-A01	PubChem 102449830
C_4300	− 9.218	**MET_793**, SER_797, ASP_800	LEU_718**,** PHE_723, **VAL_726**, **ALA_743**, **MET_790**, **LEU792**, **PRO_794, LEU_844**	*Streptomyces *sp*.* NB-A13	Pmid 30268972
C_5024	− 9.074	LEU_718, **MET_793**, SER_797, ASP_800	PHE723**, VAL_726**, **ALA_743**, **MET_790**, **LEU792, PRO_794**, **LEU_844,** LEU1001, MET1002	*Streptomyces pulveraceus*	PubChem 44271362
C_5702	− 9.183	**MET_793**, SER_797, ASP_800	LEU_718**,** PHE_723, **VAL_726**, **ALA_743**, **MET_790**, **LEU792, PRO_794, LEU_844**	*Streptomyces coelicolor* M1146	PubChem 145973966
CH7 Reference	− 6.1148	–	VAL_717, LEU_718, PHE_723, **VAL_726**, **ALA**_**743**, **MET_790**, **LEU_792**, MET_793, **PRO_794,** PHE_795, **LEU_844**	–	Pmid 32943545
OSI Co-crystallized	− 8.073	**MET_793**, **SER_797**	LEU_718, PHE_723, **VAL_726**, **ALA_743**, **MET_790**, **LEU_792**, **PRO_794,** PHE_795, TYR801, **LEU_844,** LEU1001	–	PubChem 71496458

The bold residues are the conserved one with control compounds. (†: name retrieved from StreptomeDB 3.0 database).

**Figure 2:**
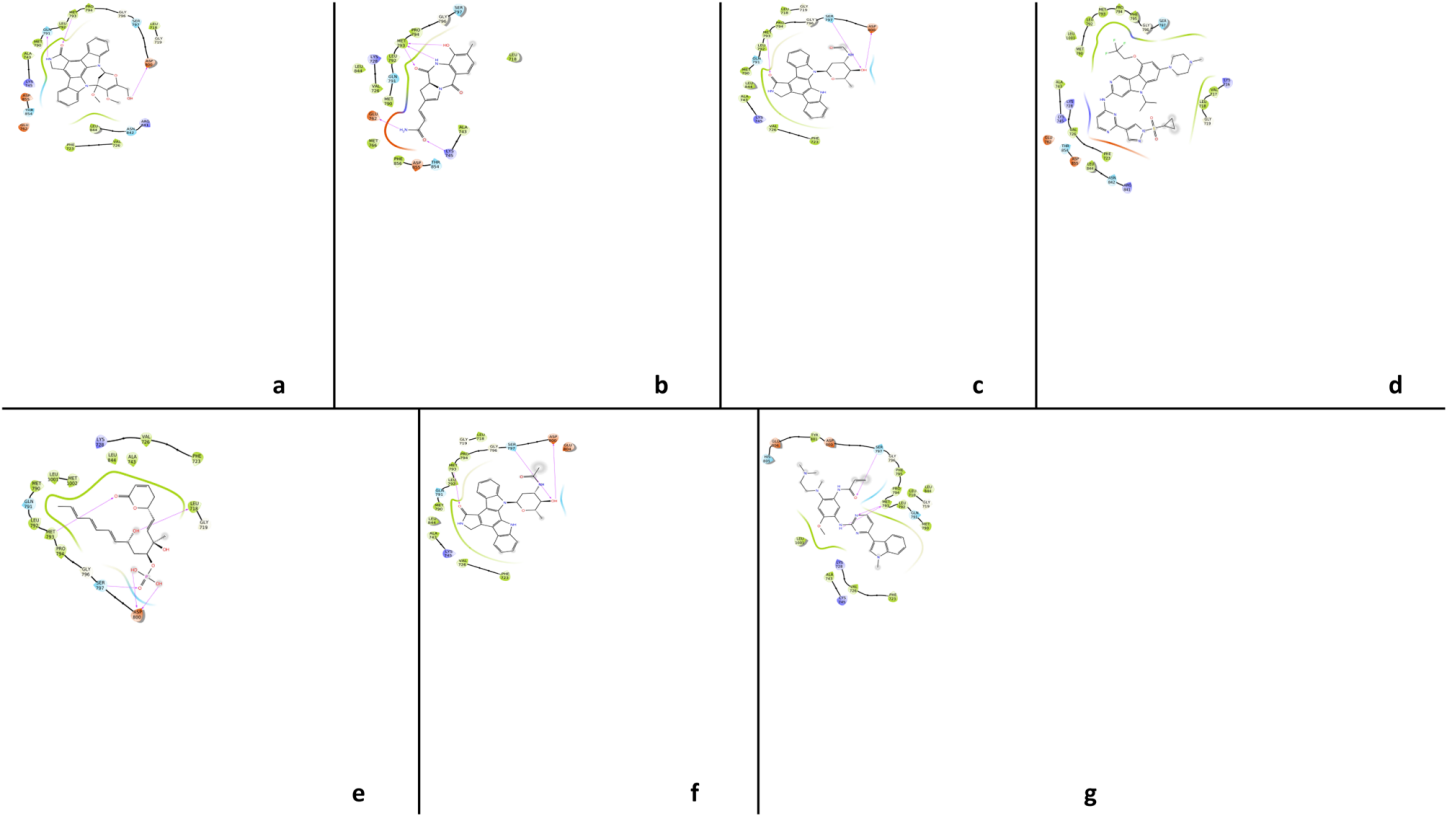
2D plot showing the network of the interaction of all compounds representing a: C_42, b: C_4299, c: C_4300, d: CH7, e: C_5024, f: C_5702 and g: Osi with active site residues of EGFR (L858R/T790M/C797S) mutant during molecular docking study.

The analysis of triple mutant EGFR^CSTMLR^ protein with interacting ligands showed that MET_793 amino acid residue forms a hydrogen bond with ligands in all five hits ligands–EGFR complex like co-crystallized ligand Osimertinib (Fig. 2). Thus, MET_793 interaction can be referred to as "vital" as it was conserved in all protein–ligands complexes^40,41^. In addition, all the protein–ligand complexes also shared the common hydrophobic bond-forming amino acid residues VAL_726, ALA_743, MET_790, LEU_792, PRO_794, and LEU_844.

Out of five screened compounds from StreptomeDB 3.0 database, ligand C42 exhibited the best docking score of − 9.3 kcal/mol.

It is a derivative of staurosporine containing carbazole ring, which made hydrogen bond with MET_793 and Gln_791 in the connecting region of C and N terminal of protein. It formed another hydrogen bond via the hydroxymethyl group of the pyrrole ring with ASP_800. The drug C_42 (Steptocarbazole A or Streptocarbazole A) was investigated for cytotoxic activity by Fu et al.^42^ and found effective against the HL-60, A-549, P388, and Hela cell line with IC_50_ values of 1.4, 5.0, 18.9, and 34.5 μM, respectively. Additionally, its inhibitory effects on the cell cycle and protein kinase were also reported. The researcher also found that Steptocarbazole A stopped the cell cycle of the Hela cell line in the G2/M phase at 10 μM concentration^42^.

Ligands C4300 and C5702 have the same binding affinities and share a common bond interaction with the EGFR receptor because of their structural similarity. Only the presence of the formyl group in C4300 and the acetyl group in C5702 distinguishes them. Both comprise a carbazole ring bonded to MET_793 and an oxane ring that interacted with the SER_797 and ASP_800 amino acid residues of the EGFR receptor through its hydroxyl group.

Carbazole moiety in C_42, C_4300, and C_5702 is a large-size, tricyclic, strong pharmacophoric moiety that provides the best binding affinity with the receptor, which leads to the downregulation of protein kinase and eventually apoptosis of cancerous cell^43^.

Zhou et al.^44^ investigated the cytotoxic activities of C_4300 (3′-*N*-formylholyrine A) on the growth of PC-3 and SW-620 cell lines with the cytotoxic activity of IC50 values 2.50 and 0.73 μM, respectively.

Xiao et al.^45^ found C_5702 (3′-*N*-acetylholyrine A) compound to have cytotoxicity activity against the tumor cell lines HCT-116, K562, and Huh 7.5 as well as the normal hepatic cell line LO2 with IC_50_ values as > 100, 32.4, 35.2, and 41.1 μM, respectively. Wang et al.^46^ also tested the same compound for inhibitory action against protein kinase C enzymes θ (PKC θ) and displayed inhibitory activity with an IC_50_ value less than 2.5 μM.

C5024 showed good interaction with protein by forming the maximum number of hydrogen bonds (four). It contains a phosphate group that interacted with SER_797 and ASP_800 residues in the ATP binding pocket, while its unsaturated lactone group made interactions in the hinge region with MET_793, which helps in forming a strong bond with protein.

Its anticancer activity is accomplished by the presence of unsaturated lactone and phosphate ester moieties^47^. It is an analog of the anticancer drug Fostriecin, which is widely known for inhibiting protein phosphatase^48,49^.

This implies that the reported compound C5024 has both phosphatase and kinase inhibitory properties, suggesting dual action. The anti-tumor properties of C5024 (CHEMBL1159781**)** have been observed by McCluskey et al.^49^ on L1210 and HCT-8 Cell Lines, with GI_50_ concentrations of 0.22 and 1.5 μg/mL, respectively.

Ligand C4299 possesses amide moieties which formed two hydrogen bonds with Glu_762 and Lys_745 amino acids and improved the binding affinity. Its benzodiazepine ring is oriented in the back of the ATP binding pocket, where it interacts with MET_793 via oxo and hydroxy group, which act as donner and acceptor and lies in the hinge region of protein kinase.

Molecular docking analysis provides only a tentative result regarding protein–ligand interaction and binding score, so before concluding, molecular dynamic simulation needed to be carried out to evaluate other factors like protein stability and compactness, solvent effects, and time.

### Molecular dynamics studies of the mutant protein

MD simulation was performed for ligands (C_42, C_4299, C_4300, 5024, and C_5702), reference drug CH7233163 and co-crystallized ligand Osimertinib to confirm their structural stability at the active site of triple mutant protein EGFR^CSTMLR^ during 1000 ns MD.

**Figure 3:**
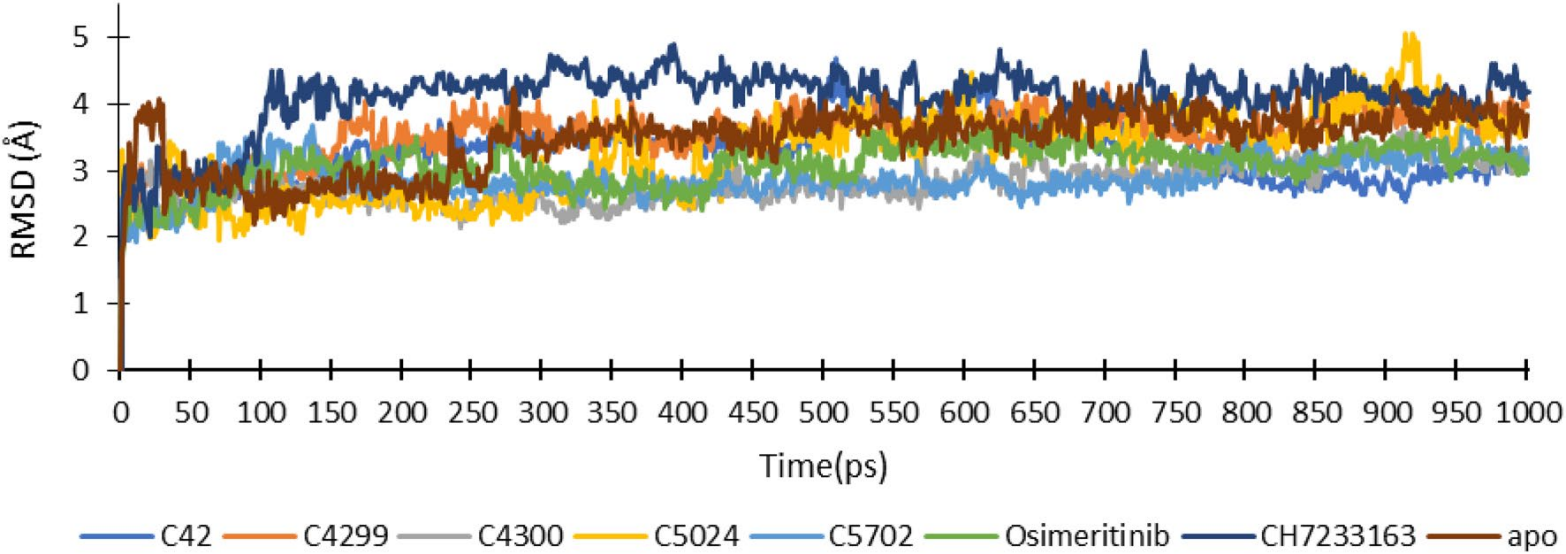
RMSD profile of triple mutant EGFR (L858R/T790M/C797S) protein complex with ligands during MD simulation of 1000 ns.

RMSD analysis of the EGFR^CSTMLR^ backbone atom confirmed that all the complexes revealed good stability (less RMSD) compared to reference inhibitor CH7233163, as shown in Fig. 3. In addition, the RMSD value for ligands C_42, C_4299, and C_5024 was comparable to co-crystallized ligand Osimertinib, while ligands C_4300 and C_5702 exhibited a lower value than Osimertinib. The average RMSD value for triple mutant EGFR + C_42, EGFR + C_4299, EGFR + C_4300, EGFR + C_5024, EGFR + C_5702, EGFR + CH7233163, EGFR + Osimertinib was 3.25 Å, 3.57 Å, 2.18 Å, 3.22 Å, 2.88 Å, 4.09 Å, 3.07 Å, respectively.

**Figure 4:**
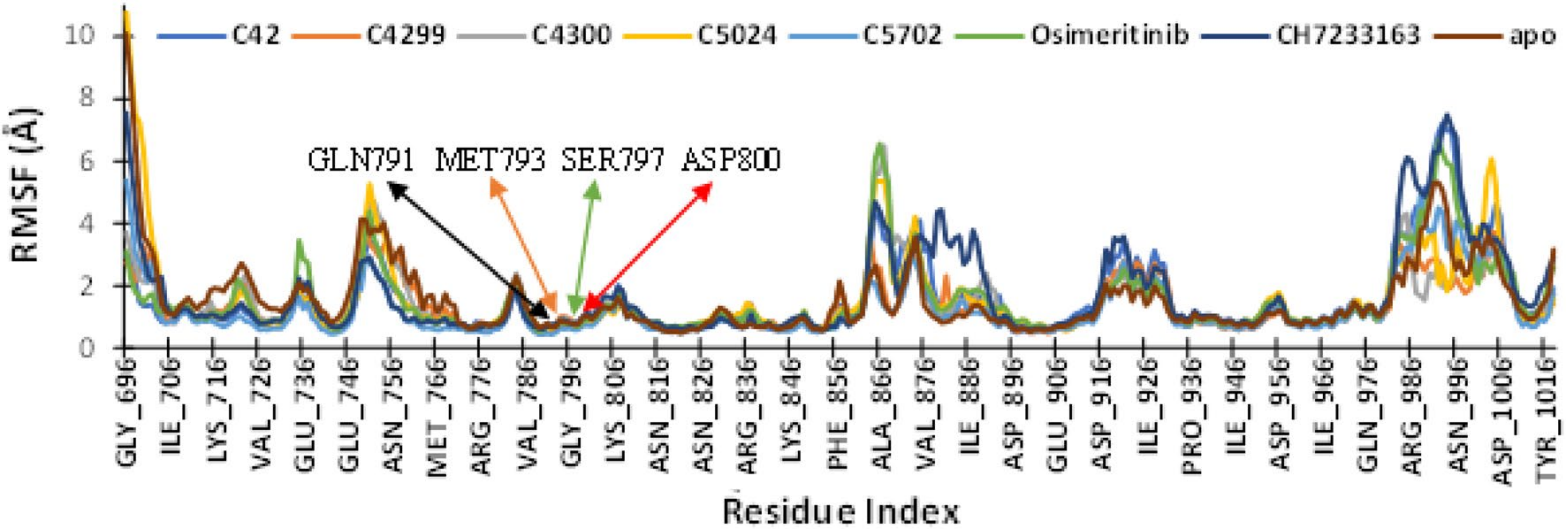
RMSF profile of triple mutant EGFR (L858R/T790M/C797S) protein’s interacting residues with ligands during MD simulation of 1000 ns.

Root mean square fluctuations (RMSF) denotes the local changes in the protein chain around the ligand. The RMSF value of all the interacting residues during the simulation is presented in Fig. 4. All the complexes revealed a comparable RMSF value with the co-crystallized ligand Osimertinib. It was found that the amino acid surrounding the ligand, GLN_791, MET_793, SER_797, and ASP_800, showed significantly less RMS fluctuations and remained stable during interactions. The average RMSF value of these amino acid residues, GLN_791, MET_793, SER_797, and ASP_800 was 0.66 Å, 0.68 Å, 0.74 Å, and 0.93 Å, respectively. The average RMSF value of ligand C_42 (1.54 Å) was very similar to the co-crystallized ligand (1.54 Å) and smaller than reference inhibitor CH7233163 (1.71 Å). It proved that protein secondary structures like alpha-helix and beta sheets remain more rigid during simulation.

**Figure 5:**
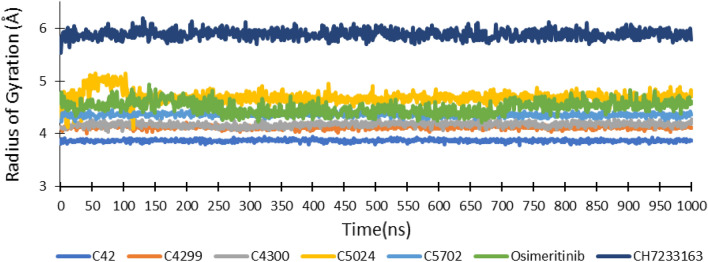
The radius of gyration of ligands during MD simulation of triple mutant EGFR(L858R/T790M/C797S) protein–receptor complex.

The R_g_ (Radius of Gyration) of ligands tells the extendedness of ligands during simulation. It was assessed that ligand C_42 comprised the lowest radius of gyration value (3.87 Å) among all ligands, co-crystallized ligand, and reference drug, as shown in Fig. 5, which illustrate that C_42 remained folded and rigid state during simulation because of its aromatic structure. R_g_ of ligand C_4299 (4.12 Å), C_4300 (4.17 Å), C_5024 (4.69 Å), and C_5702 (4.36 Å) was very comparable to co-crystallized inhibitor Osimertinib (4.49 Å). It was also observed that all ligands exhibited less R_g_ value than the reference drug CH7233163 (5.88 Å) (Fig. 5).

**Figure 6:**
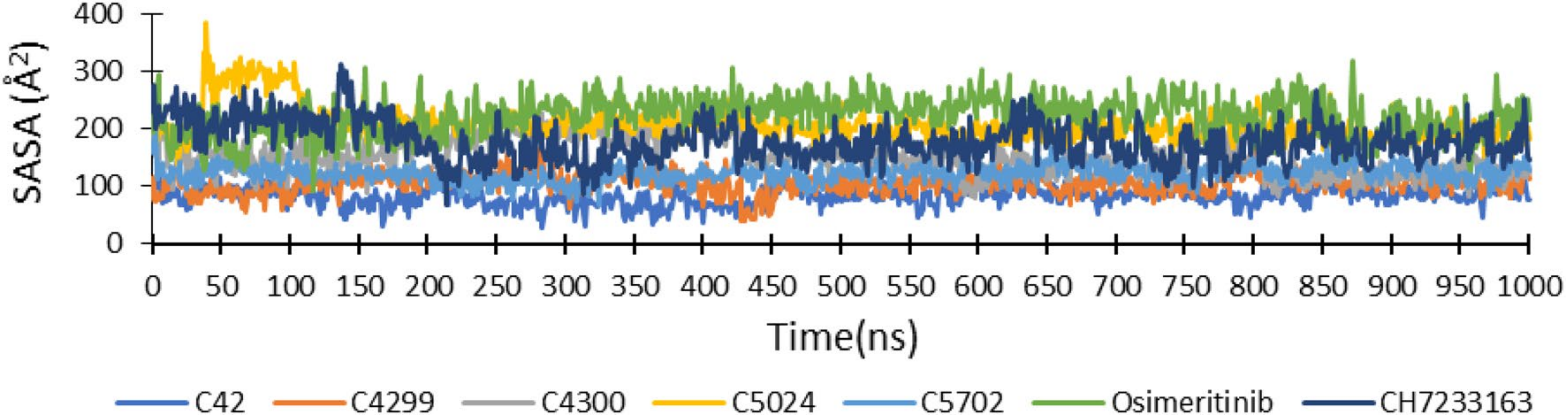
Solvent accessible surface area (SASA) of five-hit ligands, reference drug CH7233163 and co-crystallized ligand Osimertinib during MD simulation of 1000 ns.

Solvent Accessible Surface Area (SASA) evaluates the part of ligand surface area accessible by its surrounding water molecules or solvent. The plot of SASA of all ligands during MD simulation is presented in Fig. 6. The average SASA value of ligands C_42 was the lowest (82.9 Å^2^), indicating that only a tiny part of the ligand is accessible to water. Ligand C_4299 (105.79 Å^2^), C_4300 (153.44 Å^2^), and C_5702 (123.0 Å^2^) comprised less SASA value than reference drug CH7233163 (222.38 Å^2^) and co-crystallized ligand Osimertinib (178.20 Å^2^), while ligand C_5024 had higher value (208.60 Å^2^) but were comparable to the reference drug (Fig. 6).

**Figure 7:**
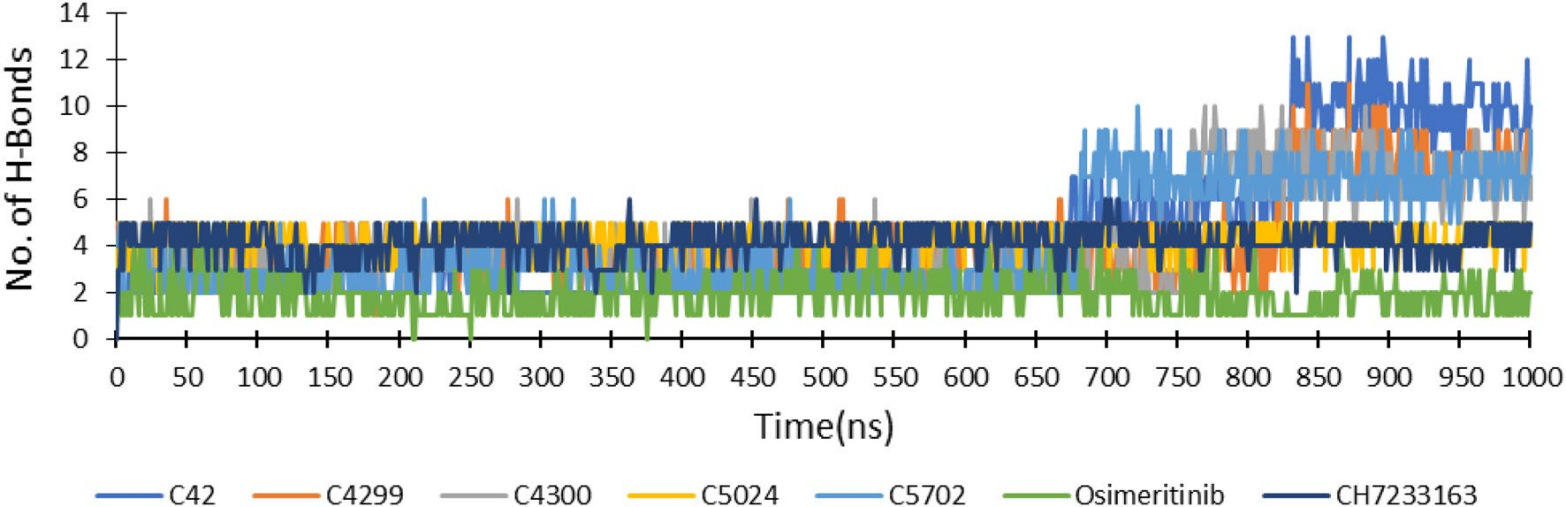
Hydrogen bonds formed between protein EGFR^L858R/T790M/C797S^ and ligands during MD simulation of 1000 ns.

The number of hydrogen bonds formed by amino acid residues with all ligands during the simulation was assessed. On average, ligands C_42, C_4299, C_4300, and C_5702 formed six hydrogen bonds till 680 ns; after that, the value was enhanced up to eleven. However, ligand C_5024 shows an average of five hydrogen bonds throughout the simulation, similar to reference drug CH7233163 and co-crystallized ligand Osimertinib, as presented in Fig. 7.

**Figure 8:**
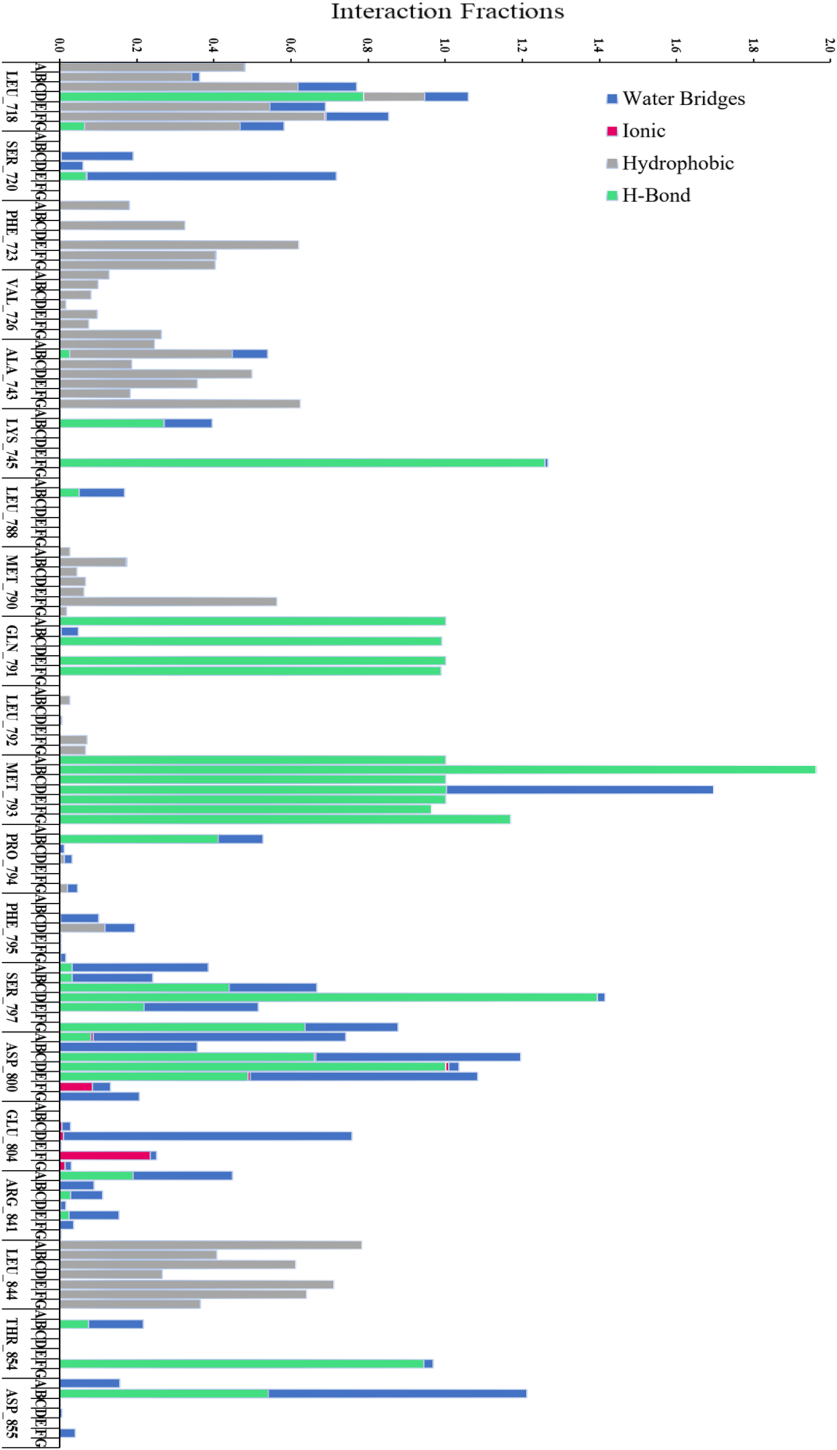
Amino acid residues contacted with ligands (A: C_42, B: C_4299, C: C_4300, D: C_5024, E: C_5702, F: CH7 and G: OSI) through molecular interaction (hydrogen-bond, hydrophobic, ionic, and water bridges) during MD simulation of 1000 ns.

MD simulation results also revealed that all the ligands form a hydrogen bond with protein residue MET_793 like reference ligand CH7233163 and co-crystallized ligand Osimertinib with 100% occupancy; in addition, ligand C_4299 remain interacted with MET_793 for the maximum period (Fig. 8).

Ligand C_42 shows hydrophobic interaction with LEU_718, PHE_723, VAL_726, ALA_743, MET_790, and LEU_844 residues, which was also seen in co-crystallized ligand Osimertinib. C_42 also showed a water bridge with a SER_797, like in the co-crystallized ligand (Fig. 8).

**Figure 9:**
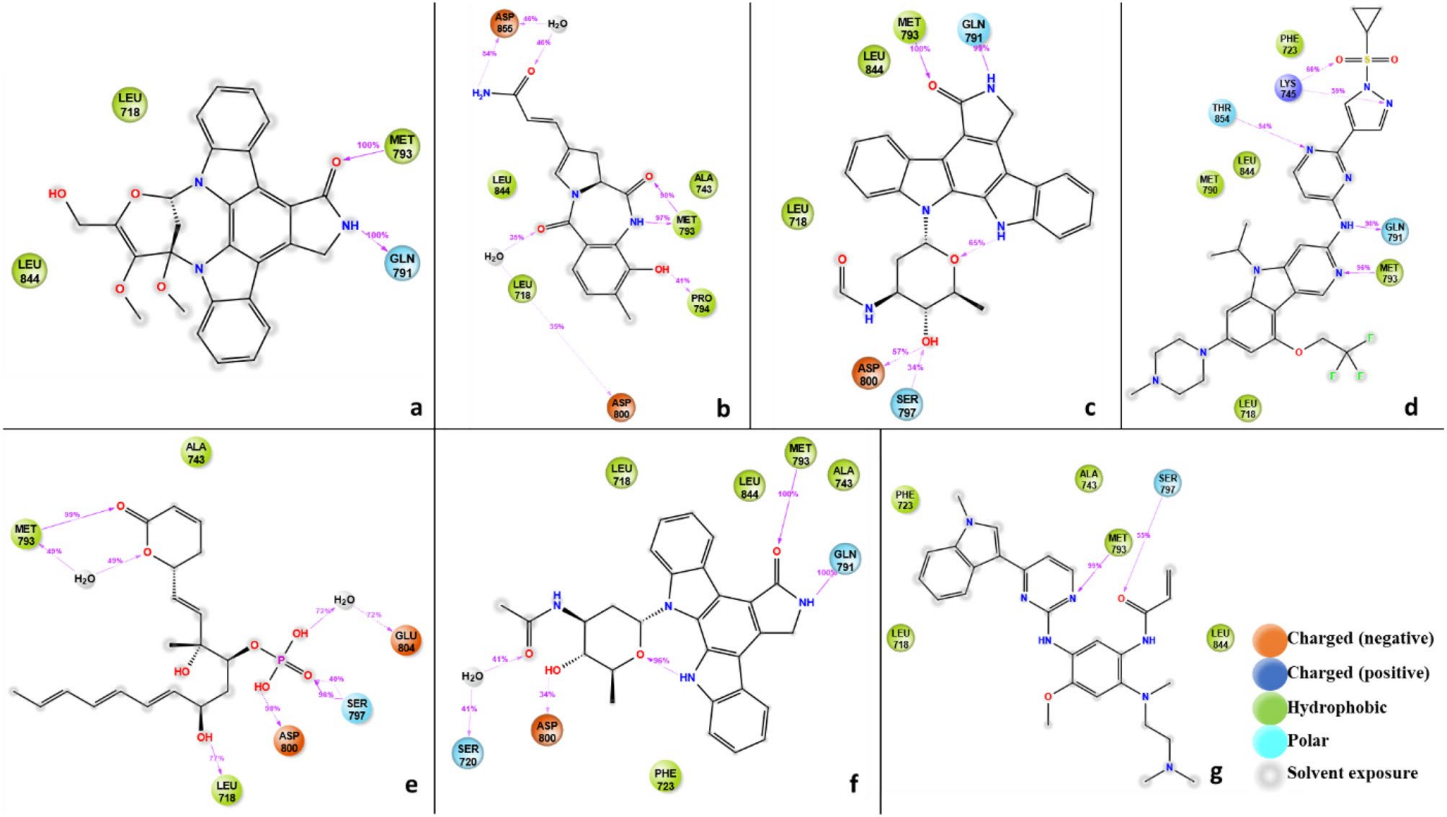
2D plot represents the ligands representing a: C_42, b: C_4299, c: C_4300, d: CH7, e: C_5024, f: C_5702 and g: Osi with interacting residues of EGFR^L858R/T790M/C797S^ and their interaction percentage during MD simulation of 1000 ns.

Ligand C_42 makes hydrogen bonds with MET_793 and Gln791 with a 100% interaction percentage, which existed in reference ligand as well with 96% and 98% interaction probability, as presented in Fig. 9. Co-crystallized ligand Osimertinib interacted with MET_793 and SER_797 residue with 99% and 55% interaction percentage, respectively (Fig. 9). Ligands C_5702, C_4299, C_4300, and C_5024 also show strong interaction with MET_793, and the interaction percentage was 97% to 100%. They also interact with ASP_800 with 34%, 35%, 57%, and 98% probability, respectively. Water-mediated interaction was also revealed in ligand C_4299 with ASP_855 and LEU_718, Ligand C_5024 with MET_793, and Ligand 5720 with Ser720 (Fig. 9).

Thus, based on molecular docking and molecular dynamic simulation results, the screened ligand C_42 exhibited the best interaction with triple mutant EGFR^CSTMLR^ protein. It comprised better binding energy, the R_g_, and SASA value than reference ligand CH7233163 and co-crystallized ligand Osimertinib. Its RMSD and RMSF values were comparable to the co-crystallized ligand and lower than the reference drug. In the beginning, it displayed four hydrogen bonds, but towards the end, it had increased to eleven. On the other hand, the co-crystallized ligand and reference drug revealed four hydrogen bonds throughout the simulation (Fig. 7).

Additionally, compared to the co-crystalized ligand, it also demonstrated conserved hydrogen bond interactions with the amino acids MET_793; water bridges with SER_797 residue; and hydrophobic interaction with LEU_718, PHE_723, VAL_726, ALA_743, MET_790, and LEU_844 residues. Thus, the overall analysis proved that ligand C_42 comprises better interaction and can be considered as an anticancer drug against triple mutant EGFR^L858R/T790M/C797S^.

### MM/GBSA calculations and per-residue free energy decomposition

With the MMGBSA technique, the free-energies of protein–ligand complexes were estimated. One hundred frames were collected from the last 100 ns of MD simulation trajectories to explore the binding capabilities of ligands with the EGFR^L858R/T790M/C797S^. The obtained results represent that the free binding energy of C_42 (− 60.06 kcal mol^−1^), C_5702 (− 61.23 kcal mol^−1^), C_5024 (− 51.24 kcal mol^−1^), and C_4300 (− 59.85 kcal mol^−1^) are better than reference drug Osimertinib (− 50.60 kcal mol^−1^) and lower but comparable to CH7 (− 68.74 kcal mol^−1^), C4299 (− 39.16 kcal mol^−1^) found relatively lower to CH7 but comparable to Osimertinib. Later on, looking at the components of free energy, it was observed that coulomb energies and electrostatic energies of inhibitors are better than CH7 drugs with a remarkable difference. In contrast, electrostatic energy values of ligands found akin to Osimertinib, also lipophilic energy contribution stays equivalent with reference drugs (Table 2). Van-der-wall energy input was found to be lower than CH7 but nearly the same as Osimertinib, but in the calculation to ΔG_bind_, coulomb and lipo-energy contribution ratio are higher in all cases than native ligands (Table 2). Therefore, it concludes that selected ligands have a great affinity toward the active site of mutant EGFR protein.

**Table 2:** Free energies (kcal/mol) of complex EGFR^L858R/T790M/C797S^ with C_42, C_4299, C_4300. C_5024, C_5702, CH7 and Osimertinib.

Ligand	Binding free energy (ΔG_bind_)	Coulomb energy (ΔE_coulomb_)	H-bond	Lipophilic energy (ΔE_lipo_)	Generalized born electrostatic solvation energy (ΔE_ele_)	Van der Waals energy (ΔE_vdw_)
C_42	− 60.06	− 18.95	− 1.11	− 15.61	24.02	− 48.60
C_4299	− 39.16	− 18.40	− 1.78	− 10.31	20.29	− 31.01
C_4300	− 59.85	− 24.20	− 1.42	− 16.08	26.84	− 45.42
C_5024	− 51.24	− 28.24	− 2.18	− 12.48	22.07	− 32.24
C_5702	− 61.23	− 24.95	− 1.55	− 16.86	28.52	− 47.40
CH7	− 68.74	− 10.17	− 1.91	− 16.69	21.47	− 64.80
Osi	− 50.60	− 16.33	− 0.80	− 14.55	27.00	− 46.80

**Figure 10:**
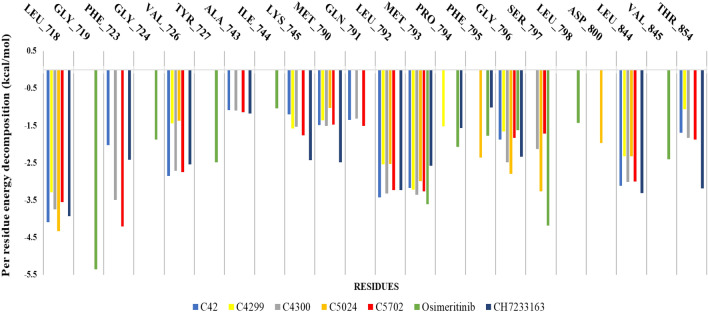
Per-residue-energy-decomposition analysis of all ligands against EGFR^L858R/T790M/C797S^ structure.

The per-residue decomposition was also performed on contributing amino acids to interpretative the energy influence profile in protein–ligand interaction (Fig. 10). The residues having free energy equal to -1 kcal mol^−1^ or lesser were taken into account to know the depth of involvement in the protein–ligand complex stability, and it found that the residues LEU_718, PHE_723, VAL_726, ALA_743, LYS_745, MET_790, LEU_792, MET_793, GLY_796, SER_797, LEU_844, and THR_854 have played an essential role in the binding of all the seven ligands. From the analysis of Fig. 10, C_5702, C_4300, and CH7 have shown making interaction with the highest number of residues, followed by C_42, then Osimertinib, and last, C_4299 and C_5024, where all the ligands, including CH7, have shared most of the residues (8–12 residues), the ligand Osimertinib found to be gone a bit out of the league with only two residues (Fig. 10).

## Conclusions

In the current research, we have attempted to identify a novel drug against triple mutant EGFR^CSTMLR^ as cancer therapy from streptomyces sources. In order to achieve this, the ADMET profile of all the streptomyces-derived compounds from the StreptomeDB database was assessed, and the screened hits were subjected to computational study. The docking result revealed five compounds, C_42, C_4299, C_4300, C_5024, and C_5702, with better binding affinity than both reference drug CH7233163 and co-crystallized ligand Osimertinib. As these drugs are derived from Actinomycetes so, their upscaled production can easily be achieved by the fermentation process. Further, the drugs shortlisted here were already validated experimentally by other researchers against several cancerous cell lines and protein kinases^42,44–46,49^. Based on the simulation study, ligand C_42 binds at the active site of EGFR^CSTMLR^ with outstanding protein stability, compactness, and solvent effects, revealing excellent inhibitory potential. While Osimertinib, a well-known anti-Tyrosine inhibitor, is chemically manufactured and has several adverse effects-developed resistance. On the other hand, C_42 derived from natural sources can be considered a potential inhibitor of triple mutant EGFR in cancer treatment and an alternative to chemically synthesized.

## Data Availability

The datasets generated during and/or analyzed during the current study are available from the corresponding author on reasonable request.
